# Improved computational epitope profiling using structural models identifies a broader diversity of antibodies that bind to the same epitope

**DOI:** 10.3389/fmolb.2023.1237621

**Published:** 2023-09-18

**Authors:** Fabian C. Spoendlin, Brennan Abanades, Matthew I. J. Raybould, Wing Ki Wong, Guy Georges, Charlotte M. Deane

**Affiliations:** ^1^ Oxford Protein Informatics Group, Department of Statistics, University of Oxford, Oxford, United Kingdom; ^2^ Large Molecule Research, Roche Pharma Research and Early Development, Roche Innovation Center Munich, Penzberg, Germany

**Keywords:** antibody, epitope profiling, structure prediction, clustering, epitope, clonotyping

## Abstract

The function of an antibody is intrinsically linked to the epitope it engages. Clonal clustering methods, based on sequence identity, are commonly used to group antibodies that will bind to the same epitope. However, such methods neglect the fact that antibodies with highly diverse sequences can exhibit similar binding site geometries and engage common epitopes. In a previous study, we described SPACE1, a method that structurally clustered antibodies in order to predict their epitopes. This methodology was limited by the inaccuracies and incomplete coverage of template-based modeling. In addition, it was only benchmarked at the level of domain-consistency on one virus class. Here, we present SPACE2, which uses the latest machine learning-based structure prediction technology combined with a novel clustering protocol, and benchmark it on binding data that have epitope-level resolution. On six diverse sets of antigen-specific antibodies, we demonstrate that SPACE2 accurately clusters antibodies that engage common epitopes and achieves far higher dataset coverage than clonal clustering and SPACE1. Furthermore, we show that the functionally consistent structural clusters identified by SPACE2 are even more diverse in sequence, genetic lineage, and species origin than those found by SPACE1. These results reiterate that structural data improve our ability to identify antibodies that bind to the same epitope, adding information to sequence-based methods, especially in datasets of antibodies from diverse sources. SPACE2 is openly available on GitHub (https://github.com/oxpig/SPACE2).

## 1 Introduction

Antibodies are important components of the adaptive immune system. An antibody recognizes foreign particles by binding to a specific site—the epitope—on their surface. As antibody function is tightly linked to the epitope it engages, studying epitopes is essential to understand immunology. For example, determining epitope specificities of antibody repertoires can increase our understanding of the immune response to disease (Tsioris et al., 2015; Bashford-Rogers et al., 2019) or differences of the immune system between individuals (Briney et al., 2019). Furthermore, epitope profiling can be applied in antibody drug discovery to identify both new binders to a desired target (Reddy et al., 2010; Zhu et al., 2013; Tsioris et al., 2015) and binders with improved affinity (Hsiao et al., 2019).

Epitopes can be determined at high resolution by solving the structure of an antibody in complex with its antigen. However, structure determination methods are too resource-intensive to be used to explore large datasets (Nilvebrant and Rockberg, 2018). Experimental epitope binning methods, such as competition assays (Abdiche et al., 2009), scale better; however, it remains difficult to analyze very large datasets as costs grow at O(n^2^) with the number of antibodies (n) to be evaluated. Competition assays also only offer low resolution as they struggle to distinguish between antibodies that bind to the same site and those that bind to distinct sites but overlap sterically.

Prior computational clustering of antibodies into functional groups that engage the same epitope can reduce the number of experiments that needs to be run or even remove the need for experimental epitope determination entirely. Most computational epitope profiling methods group antibodies based on sequence similarity. Clonotyping, the most widely used method, attempts to link antibodies that originate from the same progenitor B-cell (Greiff et al., 2015; López-Santibáñez-Jácome et al., 2019). The exact definition of a clonotype varies across the literature. Commonly, antibodies that originate from the same heavy chain V and J genes, match in CDRH3 length, and exceed a threshold CDRH3 sequence identity are considered a clonotype. Threshold values between 80% and 100% have been reported. To introduce additional leniency, the requirement for matching J genes can be neglected (Greiff et al., 2015). Clonal clustering is usually highly accurate, and antibodies within a cluster tend to engage the same epitope.

Clonotyping was originally intended to trace lineages of antibodies within an individual. Its use in functional clustering thus makes the assumption that antibodies against a given epitope must originate from progenitor B cells with shared genetic origins. However, antibodies from different lineages and with highly dissimilar sequences can adopt a similar binding site geometry and engage the same epitope (Scheid et al., 2011; Joyce et al., 2016; Rijal et al., 2019; Robinson et al., 2021; Wong et al., 2021). The ability to determine functional convergence is especially important when comparing the immune response of individuals, as different individuals exhibit personalized immunoglobulin gene usages (Briney et al., 2019). As clonotyping is not able to link antibodies from distinct genetic lineages, it loses power when analyzing antibodies originating from different sources.

Alternative methods have been developed to try and identify functionally equivalent antibodies that are not similar in sequence. Clustering antibodies by sequence similarity across predicted paratope residues can link antibodies from different clonotypes (Richardson et al., 2021). However, methods that consider structural similarity to cluster antibodies are even better suited to detect less related sequences with functional convergence because the binding site structure provides more direct evidence of antibody function than its sequence. Several methods are available that attempt to functionally link antibodies based on a representation containing structural information in addition to physicochemical properties of paratope residues (Ripoll et al., 2021; Wong et al., 2021).

In a previous study, we described the SPACE1 method (Robinson et al., 2021), which clusters antibodies based on structural similarity of homology models. The algorithm accurately clusters antibodies that bind to the same epitope and is able to functionally link antibodies with diverse sequences. However, SPACE1 is limited by the coverage of homology modeling (in the original study, only 73% of the data could be modeled to a usable standard) and its inaccuracies. The method was also only benchmarked at the level of domain consistency on one virus class. Recent progress in machine learning-based antibody structure prediction has led to more accurate structural models than those obtained with homology-based approaches, especially in cases where no template with high-sequence similarity is available (Ruffolo et al., 2020; Baek et al., 2021; Jumper et al., 2021; Abanades et al., 2022a; Ruffolo et al., 2022a; Abanades et al., 2022b; Ruffolo et al., 2022b; Lin et al., 2022). Higher accuracy and higher confidence in structural models also allow increased coverage and have the potential to improve structure-based epitope profiling.

Here, we present the Structural Profiling of Antibodies to Cluster by Epitope 2 (SPACE2) algorithm. SPACE2 builds on recent progress in machine learning-based antibody structure prediction and uses a novel clustering protocol systematically optimized and extensively benchmarked on epitope-resolution binding data. We show that SPACE2 outperforms SPACE1 by improving data coverage and identifying clusters even more diverse in sequences, genetic lineages, and species origin. These results underline that structural data, which can now be rapidly and easily generated through structure prediction tools, contain orthogonal functional information to sequence and should be considered when investigating antibody function.

## 2 Materials and methods

### 2.1 Datasets

Six datasets of antigen-specific antibodies were used to analyze SPACE2 clustering performance.

The training set on which the clustering algorithm, thresholds, and antibody region were set consisted of 3,051 antibodies against the SARS-CoV-2 receptor-binding domain (RBD). Antibodies were annotated with groups of overlapping epitopes originating from mutation escape profiling (Cao et al., 2023). We refer to this dataset as the Cao et al. (2023) training set throughout the paper.

CoV-AbDab (Raybould et al., 2021a), a dataset of anti-lysozyme antibodies, a non-public dataset of antibodies against Ebola viruses (EVs), and two non-public dataset of antibodies against non-viral targets (NVA1 and NVA2) were used as additional datasets to evaluate SPACE2. CoV-AbDab is a database of antibodies against coronavirus antigens, such as those from SARS-CoV-2, SARS-CoV-1, and MERS-CoV. A version of CoV-AbDab timestamped 3 October 2022 was used containing 10,719 antibodies with sequence data. As CoV-AbDab is a collection of antibodies reported in the literature, it contains the Cao et al. (2023) training set. When using CoV-AbDab as a test set [denoted as CoV-AbDab (test)], the training set was removed, and only the remaining 7,685 antibodies were included. Epitope data in CoV-AbDab are reported as in the original publications and range from the antigen to domain level.

A dataset of anti-lysozyme antibodies was created from all 53 lysozyme-specific antibodies in the structural antibody database (SAbDab) (Dunbar et al., 2014; Schneider et al., 2022), for which the antibody–antigen complex structure has been solved. Antibodies were grouped by their epitope using the Ab-ligity method (Wong et al., 2021) and annotated as binding to the same epitope if their Ab-ligity score was greater than a threshold of 0.1 (as in the original paper). Similarity of epitopes within an epitope group was confirmed by visual inspection.

The EV set contains 126 antibodies with epitope data ranging from antigen to domain level. The NVA1 set contains 31 antibodies with epitope data from competition assays. NVA2 contains 33 antibodies with epitope data from mutation escape profiling.

### 2.2 SPACE1

The original SPACE1 method clusters antibodies by the structural similarity of homology models. The algorithm was run as detailed in Robinson et al. (2021).

Homology models were produced using ABodyBuilder (Leem et al., 2016). ABodyBuilder uses structures from a database to build its models. In this study, we used quality-filtered SAbDab (Dunbar et al., 2014; Schneider et al., 2022) entries timestamped before 6 July 2022. Quality filtering restricts structures to those solved by X-ray crystallography and excludes structures with a resolution of >2.5 Å and structures containing residues with a B-factor >80. In a standard ABodyBuilder run, the method first attempts to model CDR loops with a template database search method (Choi and Deane, 2010). If no suitable template is found for CDRs, hybrid homology/*ab initio* modeling is performed (Leem et al., 2016). Only models for which homology templates for all six CDR loops were found are used for clustering in the SPACE1 method to keep the models as accurate as possible.

The remaining homology models are clustered by structural similarity of CDRs. The models are split into groups of antibodies with identical CDR lengths. Antibodies in each group are then clustered using a greedy clustering algorithm. The first antibody in the group is selected as the cluster center, and all antibodies with a CDR C_*α*_ RMSD smaller than a specified threshold after alignment of framework residues are added to the cluster. After all antibodies have been compared against the first cluster center, the algorithm selects the next unclustered antibody as a new cluster center, and cluster members are chosen as in the previous step. In addition to the RMSD threshold of 0.75 Å suggested by Robinson et al. (2021), we also assessed the performance at a 1.25 Å threshold.

### 2.3 SPACE2

Our novel SPACE2 algorithm clusters antibodies by the similarity of models obtained from an ML-based structure prediction tool. The method functions in four main steps. Initially, a structural model of the antibody Fv is produced using ABodyBuilder2 (Abanades et al., 2022b). ABodyBuilder2 is a deep-learning-based tool for antibody structure prediction and was trained on SAbDab structures timestamped up to 31 July 2021. The models are then split into groups of identical CDR lengths. The models in each group are then structurally aligned on the C*α* of residues in framework regions, and a pairwise distance matrix is computed of the C*α* RMSDs of CDR loop residues. The antibodies are then clustered based on these distances.

#### 2.3.1 Clustering algorithms

Eight different clustering algorithms were explored (agglomerative clustering, affinity propagation, DBSCAN, OPTICS-xi, OPTICS-DBSCAN, K-means, Butina clustering, and greedy clustering). Agglomerative clustering (Murtagh and Contreras, 2012), affinity propagation (Frey and Dueck, 2007), DBSCAN (Schubert et al., 2017), OPTICS-xi, OPTICS-DBSCAN (Ankerst et al., 1999), and K-means (MacQueen, 1967) were implemented using the scikit-learn (Pedregosa et al., 2011). Butina clustering (Butina, 1999) was implemented using the RDKit (Landrum, 2006). A greedy clustering algorithm, grouping antibodies as the algorithm described in Section 2.2, was implemented.

Parameters and evaluated ranges for each algorithm are shown in Table 1. The K-means algorithm requires an additional parameter (K) that corresponds to the predetermined number of clusters. K was set to the number of clusters obtained from agglomerative clustering using the best performing parameters.

**TABLE 1:** Clustering algorithms and parameter ranges/values evaluated during optimization.

Algorithm	Parameter	Range/values	Optimal value
Greedy clustering	RMSD threshold	0.5–10 Å	1.25 Å
Agglomerative clustering	RMSD threshold	0.5–10 Å	1.25 Å
	Linkage criterion	Complete, average, and single	Complete
Affinity propagation	Preferences	−5 to 4	Median (RMSD matrix)
DBSCAN	RMSD threshold	0.5–5 Å	1 Å
	Minimum samples	2 and 5	2
OPTICS-xi	RMSD threshold	0.5–5 Å	1.5 Å
	Minimum samples	2	2
	xi	0.005–0.5	≤0.01
OPTICS-DBSCAN	RMSD threshold	0.5–2 Å	1 Å
	Minimum samples	2	2
K-means	Initialization	Random, K-means++	K-means++
Butina clustering	RMSD threshold	0.5–5 Å	1 Å
	Reordering	True and false	False

#### 2.3.2 SPACE2-HC

A variation of the SPACE2 algorithm was implemented that clusters antibodies based on the structural similarity of heavy chains only (SPACE2-HC). The light chains were included for the modeling step, as ABodyBuilder2 (Abanades et al., 2022b) requires sequences of both chains as an input. After this step, light chains were ignored. Antibodies were grouped based on the length of the heavy chain CDRs, aligned on heavy chain framework regions, and the C_*α*_ RMSD of CDRs H1-3 calculated. Agglomerative clustering with a “complete” linkage criterion was used as the clustering algorithm of SPACE2-HC.

#### 2.3.3 SPACE2-Paratope

A second variation of the SPACE2 algorithm was implemented that clusters antibodies based on the structural similarity of CDR loops, which are predicted to form part of the paratope (SPACE2-Paratope). Structural models were produced using ABodyBuilder2 (Abanades et al., 2022b). The Paragraph method (Chinery et al., 2023) with a classifier cut-off of 0.734, as suggested in the original paper, was then used to predict residues that are part of the paratope based on the models. All CDRs containing at least one paratope residue were then labeled as paratope CDRs. Antibodies were divided into groups containing the same set of paratope CDRs. Antibodies in each group were further grouped based on the length of the paratope CDRs, aligned on heavy chain framework regions, and clustered based on the C_*α*_ RMSD of paratope CDRs. Agglomerative clustering with a “complete” linkage criterion was used as the clustering algorithm of SPACE2-Paratope.

### 2.4 Numbering scheme and region definitions

IMGT numbering (Lefranc et al., 2003) and North CDR definitions (North et al., 2011) are used throughout.

### 2.5 Analysis of structural clusters

#### 2.5.1 Domain/epitope-consistent clusters

Antibody clusters generated for the Cao et al. (2023) training set, NVA1 set, NVA2 set, and anti-lysozyme set were classified as “epitope-consistent” or “epitope-inconsistent.” “Epitope-consistent” clusters of the Cao et al. (2023) training, NVA1, and NVA2 sets only contain antibodies that bind to the same epitope group as determined by experimental epitope binning. “Epitope-consistent” clusters of the lysozyme dataset only contain antibodies that bind to the same residue-level epitope determined using crystal structures.

Owing to the lower resolution of epitopes reported in the EV set and CoV-AbDab, clusters of these datasets were classified as “domain-consistent” and “domain-inconsistent.” EV set clusters were labeled as “domain-consistent” if they only contain antibodies that engage the same antigen domain. CoV-AbDab clusters that satisfy the following rules, consistent with previous studies (Robinson et al., 2021), were determined to be “domain-consistent”:

1. Clusters that only contain antibodies that bind to the same antigen and domain.
2. Clusters that contain antibodies binding to the same domain and others that bind to the same antigen without domain-level resolution.
3. Clusters that only contain antibodies that bind to the same antigen but do not have domain-level resolution of the epitope data.
4. Clusters with internally consistent epitope data, e.g., a cluster of antibodies labeled to bind to the spike (S) protein N-terminal domain (NTD) and others labeled as S non-RBD binders, as S NTD is a subdomain of S non-RBD.

#### 2.5.2 Performance metrics

Throughout this study, we used seven metrics to analyze functional clustering. Two accuracy metrics, the fraction of epitope-consistent clusters (number of epitope-consistent multiple-occupancy clusters/number of multiple-occupancy clusters) and the fraction of clustered antibodies in epitope-consistent clusters (number of antibodies in epitope-consistent multiple-occupancy clusters/number of antibodies in multiple-occupancy clusters), were used. Two coverage metrics, the number of multiple-occupancy clusters and the number of antibodies in multiple-occupancy clusters, were used. In order to examine accuracy and coverage with one measure, we also calculated the number of antibodies in consistent multiple-occupancy clusters. Two further metrics were used to assess the diversity of antibodies within clusters: the fraction of functionally consistent clusters containing antibodies from more than one clonotype and the mean CDRH3 sequence identity within functionally consistent clusters.

#### 2.5.3 Random baseline

Random clustering was performed as a baseline. The distribution of cluster sizes obtained from the evaluated clustering algorithm with specific parameters was recorded. Clusters with an identical size distribution were then sampled randomly from the dataset, and performance metrics were calculated. Sampling was repeated 100 times, and the metrics averaged.

#### 2.5.4 Clonotyping

Clonotyping was performed using an in-house script. Lenient VH-clonotyping and Fv-clonotyping threshold conditions based on community standards were used (Greiff et al., 2015; López-Santibáñez-Jácome et al., 2019). A VH-clonotype was defined as a match in *IGHV* genes, length-matched CDRH3, and >80% CDRH3 sequence similarity. Fv-clonotypes were defined as a match in VH-clonotype, matching of IG[K/L]V genes, length-matched CDRL3, and >80% sequence identity of CDRL3.

## 3 Results

The original SPACE1 algorithm was developed to cluster antibodies by structural similarity with the aim of better identifying functional convergence. It grouped antibodies based on the structural similarity of homology models. This method was not systematically optimized and only benchmarked on a single dataset of low-resolution epitope data. Newly available ML-based structure prediction tools produce more accurate models and have better coverage than homology modeling. Here, we introduce SPACE2, which uses a state-of-the-art antibody structure prediction method and a novel clustering protocol that has been extensively optimized and then benchmarked on several datasets of high-resolution epitope data.

SPACE2 clusters antibodies in four main steps. Initially, structural models are produced using ABodyBuilder2 (Abanades et al., 2022b). The models are then separated into groups of antibodies with identical lengths of the six CDRs, followed by the computation of a pairwise distance matrix of CDR C_*α*_ RMSDs. In the final step, a clustering algorithm divides the antibodies into structural clusters. Although some loops of different lengths can adopt similar structures, we have decided to restrict structural comparison to antibodies with identical CDR lengths for the SPACE2 method as evidence suggests length-independent structural similarities are infrequent (Nowak et al., 2016; Wong et al., 2019). Restricting structural comparison to CDRs of the same length also allows for more rapid computation as RMSDs do not have to be calculated between all pairs of antibodies within the set. Optimization of the clustering protocol was performed on a training set of 3,051 antibodies against the SARS-CoV-2 receptor-binding domain (RBD) (Cao et al., 2023).

### 3.1 Evaluating an optimal clustering algorithm

**FIGURE 1:**
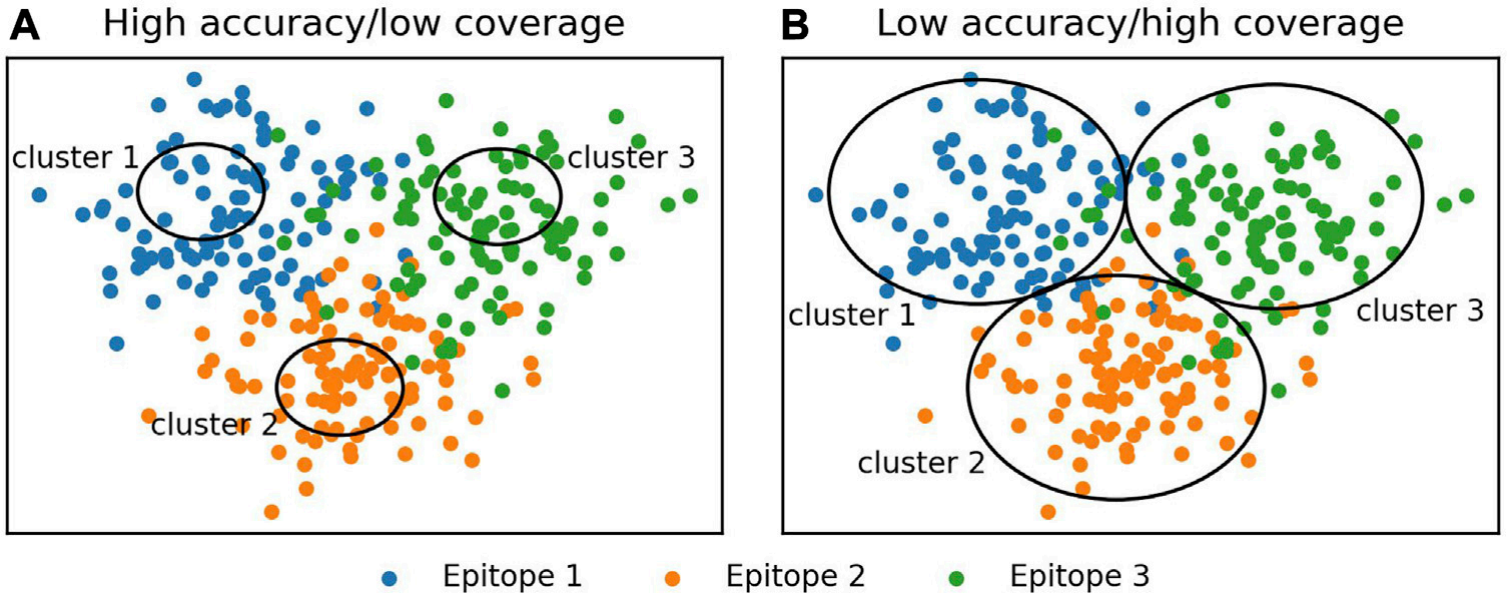
Illustration of the evaluation of clustering algorithms. Accuracy and coverage metrics were used to analyze clustering algorithms. Individually, these metrics do not necessarily indicate a good clustering algorithm, instead a trade-off between accuracy and coverage should be monitored. **(A)** High accuracy is achieved by making small clusters. These are likely to be epitope-specific; however, most antibodies are not contained in a cluster. **(B)** High coverage is achieved by the formation of large clusters. These contain most of the antibodies in the dataset but do not tend to be epitope-specific.

We tested eight widely used clustering algorithms, greedy clustering, affinity propagation (Frey and Dueck, 2007), Butina clustering (Butina, 1999), DBSCAN (Schubert et al., 2017), OPTICS-DBSCAN, OPTICS-xi (Ankerst et al., 1999), agglomerative clustering (Murtagh and Contreras, 2012), and K-means (MacQueen, 1967), for their ability to correctly group functionally consistent antibodies in the Cao et al. (2023) training set. To assess the methods, we used the number of antibodies in epitope-consistent multiple-occupancy clusters as our target performance metric as it provides a trade-off between clustering accuracy and dataset coverage. High accuracy or coverage metrics individually do not necessarily indicate a good epitope profiling method (Figure 1). High accuracy can be achieved by dividing the dataset into very small clusters that are highly likely to be epitope/domain-consistent but do not cover the full diversity of antibodies able to engage a given epitope. Maximal coverage can be achieved by putting all antibodies into a single cluster, which does not provide any useful epitope information.

**FIGURE 2:**
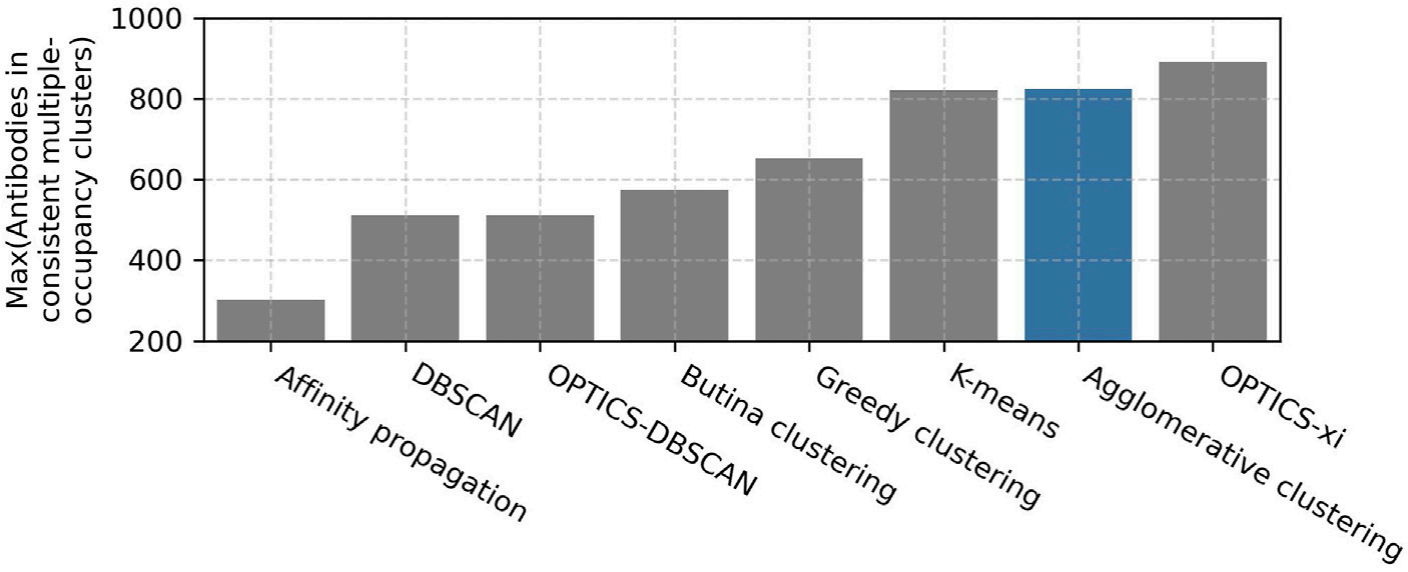
Examination of clustering algorithms. Parameter scans of eight clustering algorithms were performed using the Cao et al. (2023) training set. The performance of clustering was measured in terms of the number of antibodies in epitope-consistent multiple-occupancy clusters (*y*-axis). The maximum value of this metric achieved by a specific algorithm across all evaluated parameters when clustering the Cao et al. (2023) training set is shown. The ranges and optimal values of the evaluated parameter are shown in Table 1. The agglomerative clustering algorithm selected for SPACE2 is highlighted in blue.

A parameter scan was carried out to find the optimal setting for each clustering method. The ranges and optimal values of the evaluated parameters are shown in Table 1. As expected, lenient parameters increased dataset coverage, whereas stringent parameters improve accuracy, and the trade-off was maximized at intermediate values. The best performing algorithms, as defined by maximizing the number of antibodies in epitope-consistent multiple-occupancy clusters, were agglomerative clustering (optimal parameters: linkage criterion = complete; RMSD distance threshold = 1.25 Å), OPTICS-xi (optimal parameters: xi ≤ 0.01; RMSD distance threshold = 2 Å), and K-Means (optimal parameters: initialization method = K-means++), where K was set to the number of clusters obtained by agglomerative clustering with optimal parameters (Figure 2). As K-means does not lead to an improvement over agglomerative clustering, it was disregarded for further analysis. A visualization of the clustering obtained by the eight algorithms is shown in Supplementary Figure S1.

Agglomerative clustering and OPTICS-xi clustering were compared in more detail (Supplementary Table S1). Both algorithms achieve a similar clustering accuracy and dataset coverage. Agglomerative clustering produces larger clusters with a mean cluster size of 3.0 members and a maximum of 28 than OPTICS-xi clusters with mean 2.7 and maximum 11. When epitope-consistent clusters are larger, it suggests that they are better capturing the full diversity of the antibodies able to engage a given epitope. Therefore, agglomerative clustering was selected for use in SPACE2.

### 3.2 Examining the behavior of agglomerative clustering across different structural similarity thresholds

The RMSD threshold parameter of agglomerative clustering determines the leniency of the algorithm as it sets the maximum distance between any two antibodies in a cluster. Small thresholds restrict clustering to highly similar structures, whereas larger values allow clusters to contain more dissimilar antibodies. We evaluated agglomerative clustering for threshold values between 0.5 and 5 Å to assess how clustering results are affected.

Four metrics were monitored to assess the accuracy of clustering and dataset coverage. The fraction of epitope-consistent clusters (number of epitope-consistent multiple-occupancy clusters/number of multiple-occupancy clusters) and the fraction of clustered antibodies in epitope-consistent clusters (number of antibodies in epitope-consistent multiple-occupancy clusters/number of antibodies in multiple-occupancy clusters) were used as an accuracy measure. The number of multiple-occupancy clusters and the number of antibodies in multiple-occupancy clusters provide information on dataset coverage.

**FIGURE 3:**
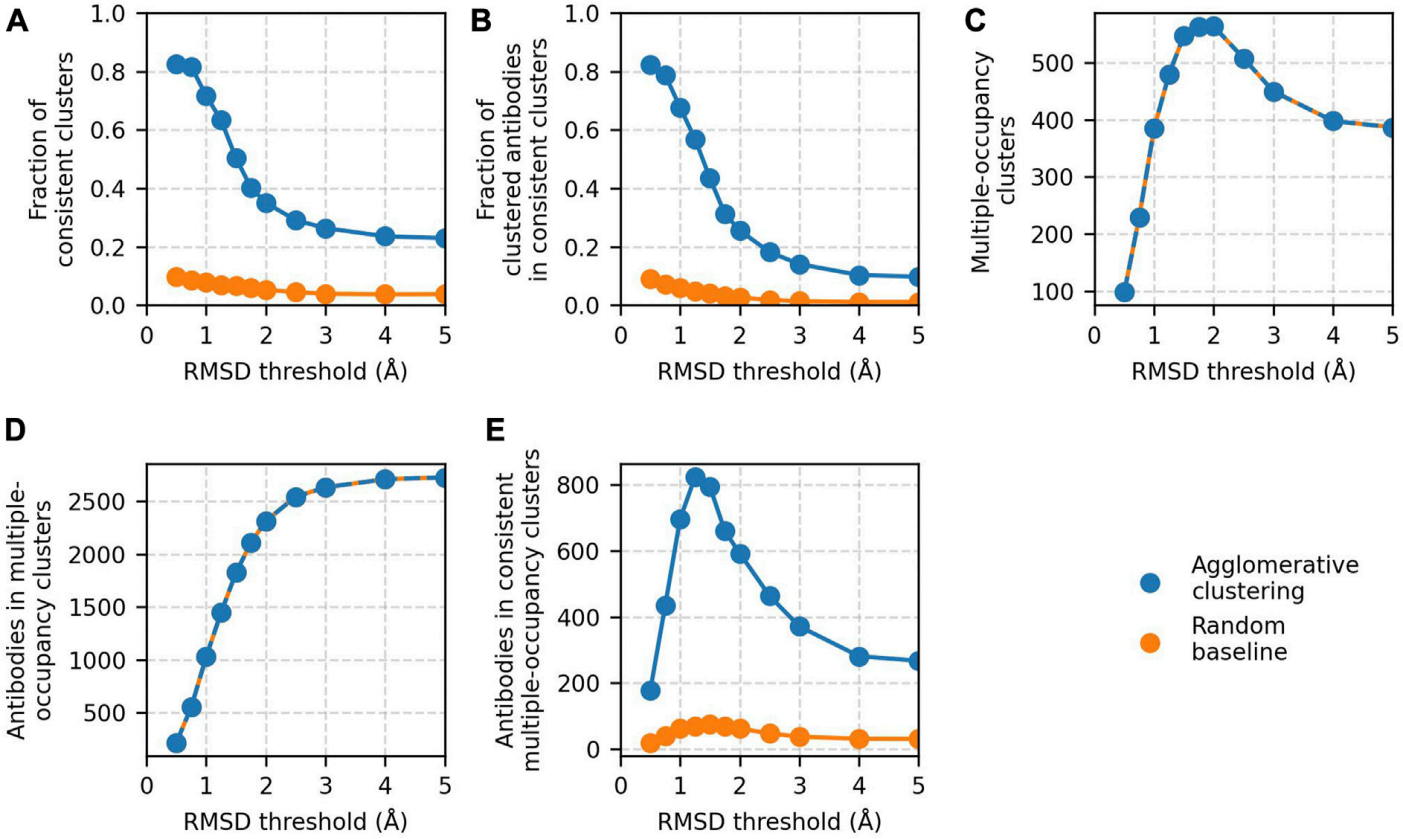
Results of agglomerative clustering as a function of RMSD threshold on the Cao et al. (2023) training set. Agglomerative clustering with a “complete” linkage criterion was performed for threshold values between 0.5 and 5 Å. The values of the five performance metrics are plotted against evaluated threshold values: **(A)** fraction of epitope-consistent clusters, **(B)** fraction of clustered antibodies in epitope-consistent clusters, **(C)** number of multiple-occupancy clusters, **(D)** number of antibodies in multiple-occupancy clusters, and **(E)** number of antibodies in epitope-consistent multiple-occupancy clusters. Results of a random clustering baseline (see Materials and Methods) are shown for comparison. Values for the number of multiple-occupancy clusters and antibodies in multiple-occupancy clusters for the random baseline are matched to agglomerative clustering.

Clustering accuracy and data coverage show a strong dependence on the RMSD threshold (Figure 3). At thresholds ≤0.75 Å, the clustering is highly accurate. More than 80% of clusters are epitope-consistent, and approximately 80% of clustered antibodies are in epitope-consistent clusters. Increasing the threshold leads to a rapid drop in accuracy but improves dataset coverage. The number of antibodies in multiple-occupancy clusters starts to plateau at approximately 3 Å. The large changes in accuracy and data coverage as a function of threshold suggest that the threshold should be adjusted depending on the aim of the epitope profiling task. Optimal clustering is achieved at a value of 1.25 Å, as defined by maximizing the number of antibodies in epitope-consistent multiple-occupancy clusters. However, the threshold can be set to any value between 0.75 and 3 Å to increase accuracy or coverage.

In all the analysis to this point, we have reported only on clusters that are 100% epitope-consistent (i.e., only contain antibodies against the same epitope). To measure the inconsistency of the remaining clusters, we analyzed the fraction of clusters in which at least 70% of the antibodies engage the same epitope. An additional 12% of clusters are >70% epitope-consistent, and these clusters contain an extra 26% of all antibodies contained in multiple-occupancy clusters (Supplementary Figure S2). This result indicates that even those clusters our standard performance metrics are marking as incorrect may contain large amounts of useful information.

### 3.3 Evaluating the optimal region for clustering

The SPACE2 method calculates structural similarity of antibodies across all six CDRs. However, not all CDRs are equally involved in binding and we expect the structure of some CDRs to be more important in determining epitope specificity than the structure of others. Therefore, we investigated how the choice of CDRs over which RMSDs are calculated impacts clustering. We assessed two variations of SPACE2 that cluster based on subsets of CDRs.

In the first variation, the algorithm was adapted to consider structural similarity of heavy chain CDRs only (SPACE2-HC). This approach was motivated by sequence-based methods, such as clonotyping, which often achieve good performance considering only the heavy-chain sequence. In SPACE2-HC, antibodies were grouped based on the length of the heavy-chain CDRs, aligned on heavy-chain framework regions, C_*α*_ RMSD of CDRs H1-3 calculated, and clustered with an agglomerative clustering algorithm with a “complete” linkage criterion. An RMSD threshold of 1.25 Å was found to optimize SPACE2-HC (Supplementary Figure S3). SPACE2-HC performed worse than the standard SPACE2 algorithm as measured by a 33% drop in the trade-off metric of antibodies in epitope-consistent clusters (Supplementary Table S2). Although SPACE2-HC slightly increased dataset coverage, a substantial decrease in accuracy was observed.

A second variation of SPACE2 was implemented to cluster antibodies based only on the similarity of CDR loops that contain paratope residues (SPACE2-Paratope). Paratope residues were predicted using the Paragraph method (Chinery et al., 2023). Models were grouped by the combination of CDR loops that contain paratope residues (paratope CDRs). The models were then grouped again based on the length of paratope CDRs, aligned on framework regions, and the C_*α*_ RMSD of paratope CDRs was calculated. A RMSD threshold of 1.5 Å was found to optimize agglomerative clustering for SPACE2-Paratope (Supplementary Figure S3). Measured by the trade-off metric, SPACE2-Paratope performed worse than standard SPACE2 (Supplementary Table S2). A slight drop in both clustering accuracy and data coverage was observed.

The best clustering results were achieved by clustering based on the structural similarity of all six CDR loops. Therefore, the standard SPACE2 method was chosen as the clustering protocol for further analysis.

### 3.4 SPACE2 performs well on sets of antibodies against diverse targets

SPACE2 was tested on five datasets of antigen-specific antibodies using the clustering algorithm (agglomerative clustering) and parameter choices (complete linkage criterion, 1.25 Å RMSD threshold) defined on the Cao et al. (2023) training set. The test sets comprised a dataset of anti-lysozyme antibodies, a non-public dataset of anti-Ebola virus antibodies, two non-public datasets of antibodies against non-viral targets (NVA1 and NVA2), and CoV-AbDab (test), a version of CoV-AbDab with training set overlap removed (see Materials and Methods) (Raybould et al., 2021a). An overview of results from clustering the test sets is shown in Table 2.

**TABLE 2:** Performance of SPACE2 on test datasets.

Dataset	Anti-lysozyme mAbs	CoV-AbDab (test)	EV	NVA1	NVA2
Antibodies in set	53	7,685	126	31	33
Fraction of antibodies modeled	1.0	1.0	0.87	1.0	1.0
Fraction of consistent clusters	1.0	0.85	0.78	0.83	1.0
Fraction of clustered antibodies in consistent clusters	1.0	0.80	0.74	0.86	1.0
Multiple-occupancy clusters	5	1,267	9	6	5
Antibodies in multiple-occupancy clusters	50 (94%)	4,188 (54%)	19 (15%)	14 (45%)	16 (48%)
Antibodies in consistent multiple-occupancy clusters	50 (94%)	3,353 (44%)	14 (11%)	12 (39%)	16 (48%)

Values of the five performance metrics and the fraction of antibodies successfully modeled using ABodyBuilder2 are shown for each dataset. For the two metrics of number of antibodies in multiple-occupancy clusters and number of antibodies in epitope-consistent multiple-occupancy clusters, a percentage is shown additionally, indicating the percentage of antibodies in the dataset. CoV-AbDab (test) denotes the subset of CoV-AbDab that is not contained in the Cao et al. (2023) training set (see Materials and Methods). CoV-AbDab (test) was used for this analysis to prevent testing on training set antibodies.

**FIGURE 4:**
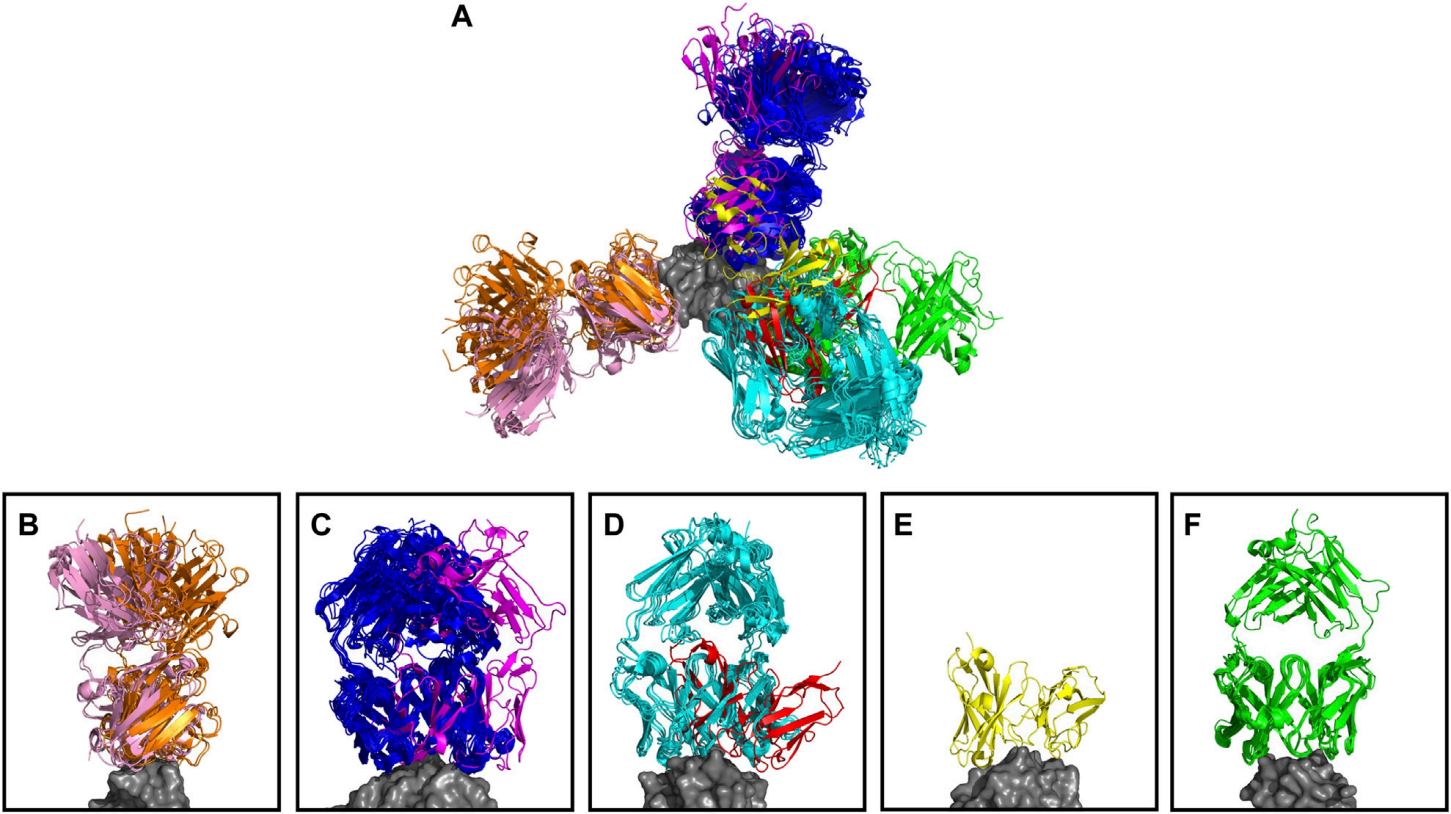
Anti-lysozyme antibodies. Crystal structures of 53 antibody–lysozyme complexes are shown aligned on the antigen structure (gray). Antibodies are colored according to the clusters assigned by SPACE2. **(A)** Overlay showing all 53 antibody–lysozyme complexes. **(B–F)** Each panel shows all antibodies that bind to one of the five lysozyme epitopes as defined by Ab-ligity (Wong et al., 2021). Panels **(B–D)** Each contain two sets of antibodies that do not overlay perfectly indicating a difference in binding pose. SPACE2 separates antibodies binding to the same epitope in a different binding pose into distinct clusters as indicated by coloring.

The anti-lysozyme dataset contains antibodies against five distinct epitopes. SPACE2 clusters antibodies in this set with high accuracy as 100% of clusters are epitope-consistent. Good data coverage is observed, and 50 of the 53 antibodies fall into multiple-occupancy clusters. SPACE2 divides the dataset into eight clusters (Figure 4). We observe three cases where antibodies binding to a common epitope are separated into two clusters. Looking at these cases in more detail shows that despite engaging the same epitope, the antibody structures do not overlay perfectly. In each case, we observe antibodies that bind to the epitope in two different binding poses, and these are separated into distinct clusters by SPACE2. These results show that SPACE2 groups antibodies with a high resolution.

SPACE2 also achieves a high clustering accuracy on CoVAbDab (test), the EV set, the NVA1 set, and the NVA2 set; 85%, 78%, 83%, and 100% of clusters in the four sets are domain/epitope-consistent, respectively. Domain/epitope-consistent clusters comprise 80%, 74%, 86%, and 100% of all antibodies grouped into multiple-occupancy clusters in the four sets, respectively.

Data coverage differs for the four datasets (see Materials and Methods for definition of coverage metrics). Coverage of CoVAbDab (test), the NVA1 set, and the NVA2 set is high, with 54%, 45%, and 48% of all antibodies contained within multiple-occupancy clusters, respectivelwhich balances both accuracy and cy. In comparison, only 19 of 126 EV set antibodies are grouped into multiple-occupancy clusters. The EV set is relatively small and contains antibodies against the Ebola virus glycoprotein, a large multi-domain protein with many potential epitopes (Lee et al., 2008). We do not expect to observe many antibodies engaging the same residue-level epitope in a small dataset of antibodies against a target with many epitopes, which is likely why we see low coverage.

Antibodies within the same epitope group in CoV-AbDab, the EV set, the NVA1 set, and the NVA2 set tend to be split across a large number of SPACE2 clusters. This is explained by the low resolution of epitope labels in these datasets and antibodies annotated with the same epitope label likely bind to a large number of different residue-level epitopes.

Overall, SPACE2 generalizes well to the test sets. The algorithm achieves a high clustering accuracy on all five datasets and a good coverage on CoV-AbDab (test), NVA1, NVA2, and anti-lysozyme datasets. Coverage of the EV set is comparably low, indicating a challenge in clustering smaller datasets of epitope-diverse antibodies.

### 3.5 Advances in structure prediction improve structure-based computational epitope profiling

We compared the performance of SPACE2 to SPACE1, our previous structural epitope profiling method. SPACE1 (Robinson et al., 2021) groups antibodies based on structural similarity of homology models produced using ABodyBuilder (Leem et al., 2016) followed by greedy structural clustering at an RMSD threshold of 0.75 Å.

We, once again, used the number of antibodies in epitope/domain-consistent multiple-occupancy clusters, which balances both accuracy and coverage, as our metric for comparing performance. SPACE2 outperforms SPACE1 using its suggested threshold (RMSD threshold 0.75 Å) (Supplementary Table S3). As SPACE2 uses an RMSD threshold of 1.25 Å, we also explored a range of RMSD values to see whether the difference in the RMSD threshold is the driver for the difference in performance. We found that a threshold of 1.25 Å improved SPACE1 clustering (Supplementary Table S3), but it was still significantly worse than SPACE2. SPACE1 with a 1.25 Å threshold results in an 18% and 9% decrease in antibodies in epitope/domain-consistent multiple-occupancy clusters on the two largest datasets, CoV-AbDab and the Cao et al. (2023) training set, respectively (Table 3). SPACE2’s better performance is driven by better coverage while achieving a similar accuracy.

**TABLE 3:** Comparison of SPACE2, SPACE1, and clonotyping.

Dataset	CoV-AbDab				Training set			
Method	SPACE2	SPACE1	Clonotyping		SPACE2	SPACE1	Clonotyping	
			VH	Fv			VH	Fv
Fraction of antibodies modeled	**1.0**	0.71	-	-	**1.0**	0.7	-	-
Fraction of consistent clusters	0.87	0.87	0.98	**0.99**	0.63	0.64	0.84	**0.83**
Fraction of clustered antibodies in consistent clusters	0.82	0.81	0.97	**0.99**	0.57	0.58	0.75	**0.79**
Multiple-occupancy clusters	**1,811**	1,165	1,191	970	**480**	314	361	303
Antibodies in multiple-occupancy clusters	**6,271 (59%)**	4,010 (37%)	4,045 (38%)	2,754 (26%)	**1,446 (47%)**	935 (31%)	1,060 (35%)	793 (26%)
Antibodies in consistent multiple-occupancy clusters	**5,126 (48%)**	3,255 (30%)	3,916 (37%)	2,733 (25%)	**823 (27%)**	538 (18%)	797 (26%)	628 (21%)
Fraction of clusters containing >1 VH-clonotypes	**0.81**	0.71	0	0	0.55	**0.58**	0	0
Mean CDRH3 sequence identity	**0.54**	0.57	0.88	0.88	**0.66**	0.67	0.86	0.87

The original SPACE1 algorithm was evaluated at an RMSD threshold of 1.25 Å. Two protocols were used for clonotyping (see Materials and Methods). VH-clonotyping is restricted to genes and sequence of the heavy chain. Fv-clonotyping considers both heavy and light chains. For the two metrics of number of antibodies in multiple-occupancy clusters and number antibodies in epitope-consistent multiple occupancy clusters, a percentage is shown additionally, indicating the percentage of antibodies in the dataset. The most important performance metric to consider when comparing different epitope profiling methods is the number of antibodies in epitope/domain-consistent multiple-occupancy clusters as high accuracy or coverage metrics individually may not indicate good performance. The fraction of epitope/domain-consistent clusters containing more than one VH-clonotype and the mean CDRH3 sequence identity observed within epitope/domain-consistent clusters are also given. The best result for each metric is highlighted in bold.

**FIGURE 5:**
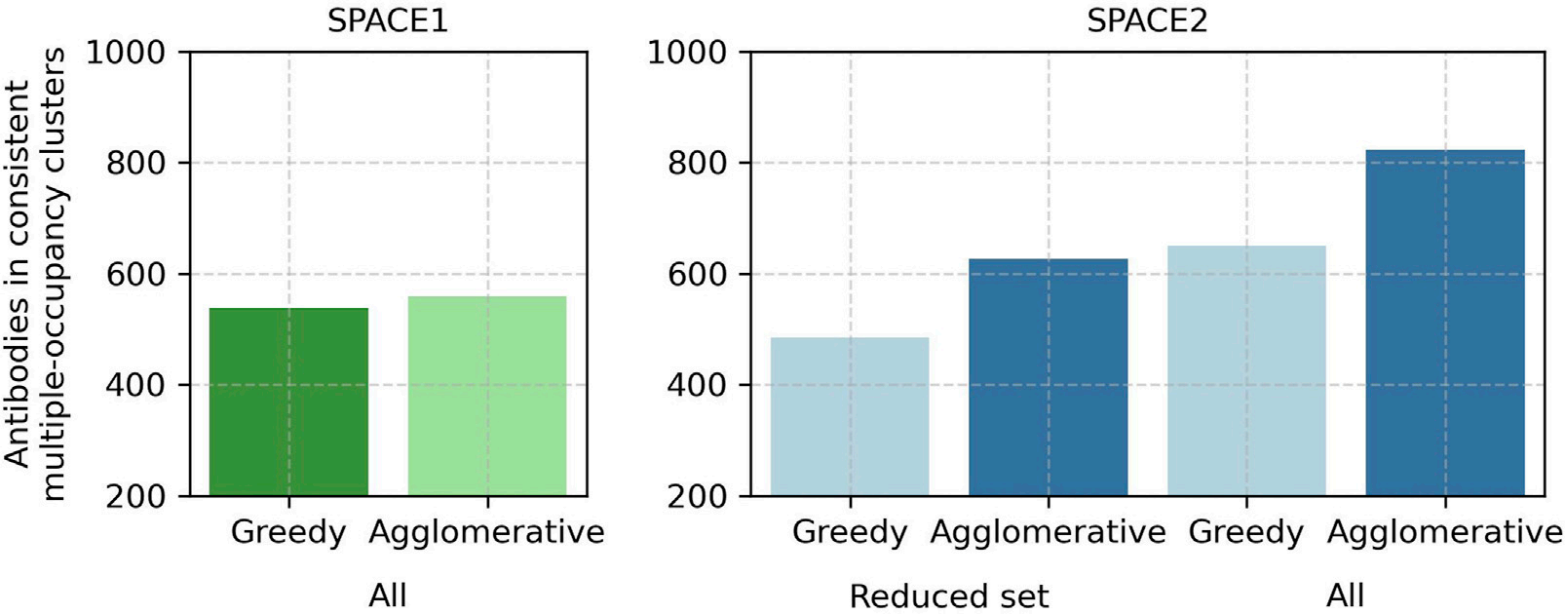
In-depth comparison of SPACE2 and SPACE1 performance on the Cao et al. (2023) training set. SPACE1 with a 1.25 Å RMSD threshold (green) and SPACE2 (blue) as well as an adaptation of SPACE1 (light green) using the default agglomerative clustering algorithm of SPACE2 (complete linkage criterion, 1.25 Å RMSD threshold) and an adaptation of SPACE2 (light blue) using the default greedy clustering algorithm of SPACE1 (1.25 Å RMSD threshold) were evaluated on the complete data set (all). Additionally, SPACE2 and its adaptation were evaluated on a reduced dataset which only included the 2,140 antibodies successfully modeled by homology modeling (reduced set).

Modifications of the SPACE2 and SPACE1 methods reveal that the better performance of SPACE2 arises due to the larger number of antibodies modeled with ML-based structure prediction compared to homology modeling and better clustering with the agglomerative clustering protocol compared to greedy clustering. The higher quality of models obtained from ML-based structure prediction does not lead to clear improvements in clustering (Figure 5; Supplementary Table S4).

### 3.6 SPACE2 improves coverage compared to clonotyping

Clonotyping is the most commonly used epitope profiling method. It clusters antibodies based on sequence similarity. As clonotyping assumes that antibodies against a given epitope must originate from progenitor B cells with shared genetic origins, it cannot detect functional convergence. Thus, the method is limited when clustering datasets of antibodies from different individuals or species. Here, we compare SPACE2 to two lenient clonotyping protocols, VH- and Fv-clonotyping (see Materials and Methods), on the two largest datasets which contain antibodies from diverse sources. The Cao et al. (2023) training set consists of antibodies isolated from 165 human patients (Cao et al., 2023), and CoV-AbDab contains antibodies from a range of studies (∼450) and several species (Raybould et al., 2021a).

The performance of SPACE2 and the two clonotyping protocols are shown in Table 3. SPACE2 outperforms both VH- and Fv-clonotyping in the key metric of antibodies in epitope/domain-consistent clusters on both datasets. Improvement in this metric is driven by increased dataset coverage by SPACE2. We observe 33% and 21% more antibodies in multiple-occupancy clusters for Fv-clonotyping of CoV-AbDab and the Cao et al. (2023) training set, respectively. Data coverage by VH-clonotyping is better but still substantially lower than SPACE2. On the other hand, SPACE2 is less accurate than both clonotyping protocols. However, the increase in coverage achieved by SPACE2 exceeds the drop in accuracy.

### 3.7 SPACE2 identifies functional convergence signals

We next analyzed the diversity of antibodies within the SPACE2 clusters of the Cao et al. (2023) training set and CoV-AbDab to see whether we were identifying functionally similar antibodies with very different sequences.

The majority of SPACE2 clusters contain antibodies belonging to several clonotypes, highlighting the ability to link antibodies from different genetic lineages. Specifically, 55% of epitope-consistent clusters from the Cao et al. (2023) training set and 81% of domain-consistent clusters from CoV-AbDab contain antibodies from more than one VH-clonotype (Table 3).

Moreover, we investigated the sequence similarity of antibodies within epitope/domain-consistent clusters. Clonotyping is limited to linking sequence-similar antibodies as the method uses a CDRH3 sequence identity cutoff to cluster antibodies. Here, we use a lenient cutoff of 80%. We observed a mean CDRH3 sequence identity of 86% within epitope-consistent VH-clonotypes of the Cao et al. (2023) training set and 88% for domain-consistent VH-clonotypes of CoV-AbDab. In comparison, SPACE2 clusters tend to be highly diverse in sequence. Epitope/domain-consistent clusters have a mean CDRH3 sequence identity of 54% and 66% for CoV-AbDab and the Cao et al. (2023) training set, respectively (Table 3). A large number of CoV-AbDab clusters were observed with a mean sequence identity below 40% (Supplementary Figure S4), and some clusters even contain pairs of antibodies with no common CDRH3 residues.

Structural clustering is also able to functionally link antibodies from different organisms. For CoV-AbDab, SPACE2 produced 26 functionally consistent clusters containing antibodies from more than one species and was able to group antibodies from human, mouse, and rhesus macaque origins. In comparison, optimized SPACE1 was only able to detect 18 domain-consistent inter-species clusters, and clonotyping is unable to link antibodies from different species.

#### 3.7.1 SPACE2 informs on functional convergence of sequence-dissimilar antibodies

**FIGURE 6:**
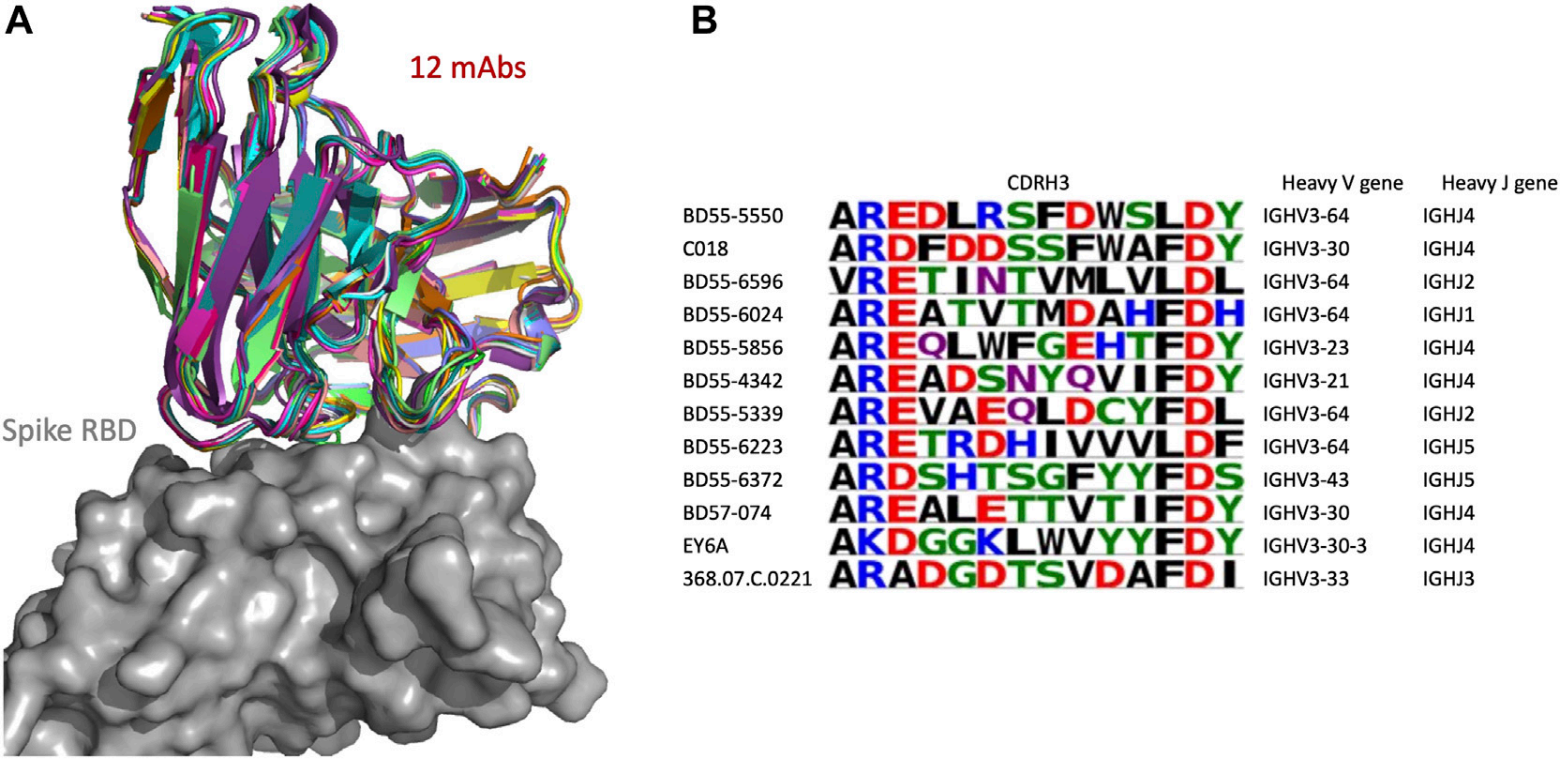
SPACE2 identifies functional convergence of antibodies. Twelve-membered CoV-AbDab cluster (368.07.C.0221, BD55-4342, BD55-5339, BD55-5550, BD55-5856, BD55-6024, BD55-6223, BD55-6372, BD55-6596, BD57-074, C018, and EY6A) with a mean CDRH3 sequence identity of 33%. The crystal structure of EY6A in complex with its antigen is available (PDB 6ZCZ). SPACE2 suggests that the 11 remaining antibodies bind to the same residue-level epitope. **(A)** Structural models of the 12 members are overlaid with the crystal structure of EY6A (not shown) in complex with the spike protein RBD (gray). **(B)** CDRH3 sequence alignment of all 12 cluster members colored by chemical properties of amino acid residues [produced with Logomaker (Tareen and Kinney, 2020)] and heavy-chain V and J genes.

We examined in more detail a SPACE2 cluster of the CoV-AbDab dataset with 12 members (368.07.C.0221, BD55-4342, BD55-5339, BD55-5550, BD55-5856, BD55-6024, BD55-6223, BD55-6372, BD55-6596, BD57-074, C018, and EY6A) (Figure 6). Eleven of the antibodies engage the spike protein RBD, and the final member is annotated as a spike protein binder with an unknown domain. Clustering by SPACE2 suggests that these antibodies, determined to bind to the same domain of the spike protein, engage the same residue-level epitope.

The 12 antibodies are highly diverse in sequence and genetic lineage. The cluster shows a mean CDRH3 sequence identity of 33%. The antibodies possess a CDRH3 of length 12, and eight of these residues differ on average. The most distant pair of antibodies in the cluster is BD55-6596 and EY6A, which differ in 11 of 12 CDRH3 residues. The 12 antibodies originate from seven different IgGH genes and fall into 12 separate VH-clonotypes.

The improvement of SPACE2 over SPACE1 can be seen when examining how these antibodies were clustered by SPACE1. Using SPACE1 with an optimized threshold, only six of these antibodies (BD55-6024, BD55-6223, BD55-6596, BD57-074, C018, and EY6A) were grouped together, and even these six were a part of a larger functionally inconsistent cluster with 44 members. BD55-5339 was in a separate functionally inconsistent SPACE1 cluster with four members, and the remaining five antibodies were not placed in multiple-occupancy clusters.

#### 3.7.2 SPACE2 identifies epitopes targeted by multiple species

**FIGURE 7:**
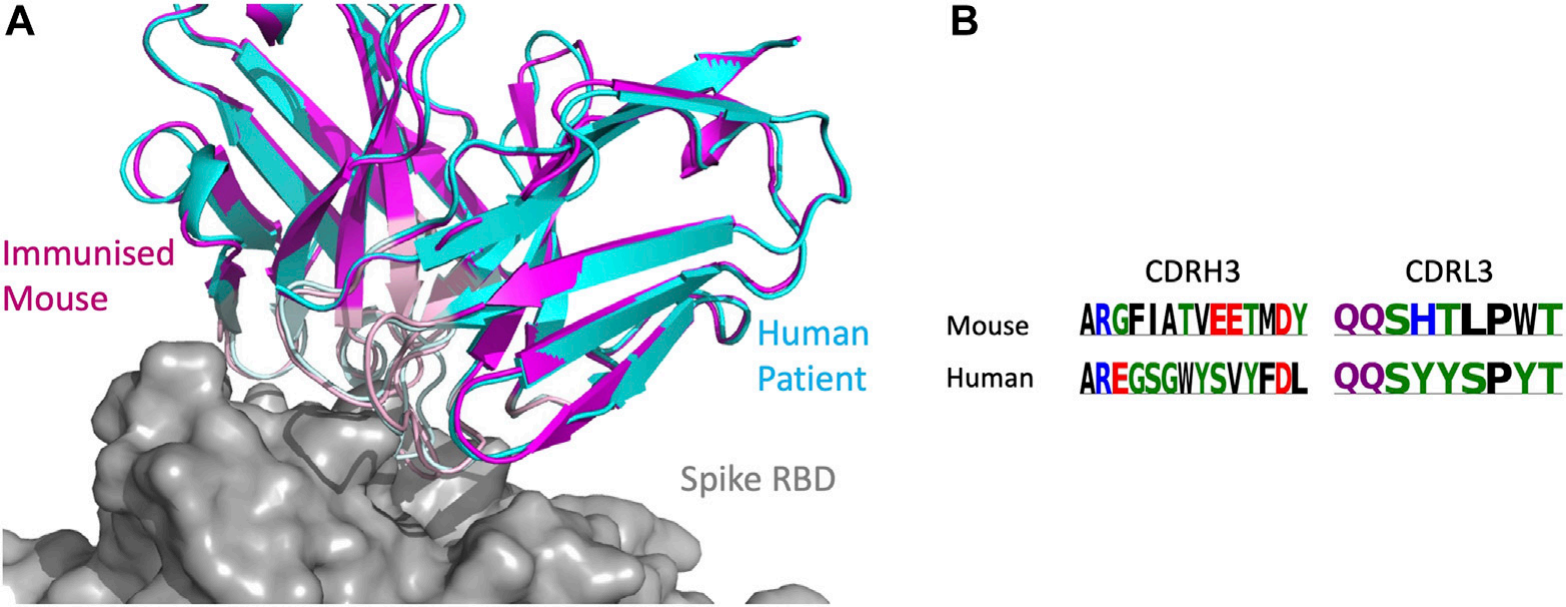
SPACE2 identifies epitopes targeted by multiple species. Two representatives from a SPACE2 CoV-AbDab cluster comprising murine (SW186) and human (BD57-092) antibodies are shown. The crystal structure of SW186 is available (PDB 8DT3). SPACE2 suggests that BD57-092 binds to the same residue-level epitope. **(A)** Structural models of SW186 (pink) and BD57-092 (cyan) were overlaid with the crystal structure of SW186 (not shown) in complex with the spike protein RBD (gray). CDR regions of both antibodies are highlighted by lighter coloring. **(B)** CDRH3 sequence alignment of the two antibodies colored by chemical properties of amino acid residues [produced with Logomaker (Tareen and Kinney, 2020)].

As the CoV-AbDab database contains antibodies from multiple species (human, mouse, and rhesus macaque), we examined whether SPACE2 can identify epitopes targeted by multiple species. There were 26 SPACE2 clusters of the CoV-AbDab database that contained antibodies from more than one species. We examined a SPACE2 cluster with seven members (368.02a.C.0049, B13, BD55-6574, BD57-092, DK15, Fab-160, and SW186) (Figure 7). The cluster contains six antibodies that engage the spike protein RBD and one spike-specific antibody without domain-level epitope data. Five of the antibodies have human genetics and originate from human patients, phage-display, and transgenic mice. The remaining antibodies have murine genetics and were raised by immunized mice. SPACE2 suggests that these genetically human and mouse antibodies engage the same residue-level epitope which highlights its ability to detect public epitope targeted by multiple species.

SPACE1 with an optimized threshold was only able to link one of the two murine antibodies in this cluster to human structures, while clonotyping is unable to link mouse and human antibodies due to different gene usage.

## 4 Discussion

Accurately identifying the epitope of antibodies is a key step in understanding immunology and in the design of new biological drugs. Such data are conventionally determined experimentally either by solving individual antibody–antigen crystal structures or by epitope binning methods, such as competition binding assays. Prior computational clustering of antibodies into functional groups could reduce the number of experiments that needs to be carried out or even remove the need for them entirely. Clonal clustering is most commonly used for this purpose, where antibodies are grouped by sequence identity and genetic lineage. However, these types of methods will miss antibodies with low sequence identity that have functionally converged and target common epitopes (Scheid et al., 2011; Joyce et al., 2016; Rijal et al., 2019; Robinson et al., 2021; Wong et al., 2021; Shrock et al., 2023).

In a previous study, we reported the SPACE1 method (Robinson et al., 2021), which clusters antibodies by structural similarity of their homology models. This method showed that structure-based epitope profiling is better able to detect the full breadth of functional convergence. However, SPACE1 was limited by the coverage of template-based modeling and its inaccuracies. The method was also only benchmarked at the level of domain-consistency of antibodies against one virus class. Here, we introduce SPACE2, an updated method which uses the latest machine learning-based antibody structure prediction technology (Abanades et al., 2022b) and a novel clustering protocol systematically optimized on epitope-resolution data.

We show across six datasets that SPACE2 can accurately bin antibodies that engage the same epitope and achieve high data coverage. Available crystal structures of antibody–antigen complexes reveal that SPACE2 tends to group antibodies that bind to the same residue-level epitope in an identical binding pose. Epitope resolution of SPACE2 appears to be similar to that obtained from crystal structures and higher than data from epitope binning methods which struggle to distinguish between antibodies that bind to the same site and those that bind to distinct sites but overlap sterically.

SPACE2 outperforms our previous epitope profiling tool SPACE1 (Robinson et al., 2021) and clonotyping when considering the number of antibodies in epitope-consistent multiple-occupancy clusters. Clonotyping is more accurate than SPACE2 but has far lower coverage. The lower accuracy of SPACE2 is explained by the fact that antibodies with similar CDR structures may engage different epitopes if chemical properties of the CDR residues are significantly different.

We also highlight how our methodology allows the detection of functional convergence across populations of antibodies. Across functionally consistent clusters of our largest dataset, CoV-AbDab (Raybould et al., 2021a), we detect a mean CDRH3 sequence identity as low as 54%. Furthermore, we observe 26 functional clusters containing antibodies from multiple species including human, mouse, and rhesus macaque antibodies. In comparison, sequence-based epitope profiling such as clonotyping is severely restricted in grouping sequence-diverse antibodies and is not able to link antibodies from different genetic origins and species (Greiff et al., 2015; López-Santibáñez-Jácome et al., 2019; Raybould et al., 2021b). Although it is possible to cluster nanobodies with the SPACE2-HC implementation, we were unable to detect functional convergence to antibodies when testing on CoV-AbDab. No clusters were detected containing both antibodies and nanobodies suggesting that the two formats use different binding site structures to engage common epitopes.

SPACE2 clusters antibodies based on the length and structural similarity of all six CDRs. This approach may constrain the detection of functional convergence to some extent as it assumes that antibodies require the same length of all six CDRs to engage the same epitope. We tried to address this issue by evaluating two adaptations of SPACE2 that reduce the number of CDRs required to have the same length. An implementation clustering antibodies based on heavy-chain structural similarity (SPACE2-HC) caused a drastic decrease in clustering accuracy. This indicates that light chain structures are important for determining antibody binding specificity, which is in line with previous findings on the functional selection of light chains (Jaffe et al., 2022) and their structural importance (Guloglu and Deane, 2023). Similarly, combining SPACE2 with information from paratope prediction (SPACE2-Paratope) (Chinery et al., 2023) and computing structural similarity only across CDRs predicted to contain paratope residues currently led to fewer functionally consistent clusters. Furthermore, some loops of different lengths can adopt similar structures (Nowak et al., 2016; Wong et al., 2019). Although evidence suggests that this is infrequent, future work could focus on being able to detect functional convergence across different CDR lengths.

The ability to detect functional convergence of antibodies will provide valuable insights into the humoral immune response. SPACE2 is able to provide more complete information on public epitopes targeted by antibodies originating from different individuals and species. Although previous studies show several public epitopes are largely distinct between species (Shrock et al., 2023), here, we identify a number of inter-species clusters. Structural clustering of larger datasets of antibodies isolated from various species will further improve our understanding of differences in their immune responses.

Although SPACE2 is computationally more expensive than sequence-based epitope profiling, it is tractable for datasets of 10^4^ antibodies, a typical number of sequences obtained from methods such as 10× sequencing (Supplementary Figure S5). The rate limiting step of SPACE2 is currently the prediction of antibody structures. Improvements in the speed of structure prediction tools as well as the release of antibody databases containing pre-modeled structures (Abanades et al., 2022b) will contribute to reducing the computational cost of structure-based epitope profiling.

Overall, SPACE2 efficiently detects functional convergence of antibodies with highly diverse sequences, genetic lineage, and species origins, further illustrating that predicted structures should be considered when investigating the function of antibodies. SPACE2 is openly available on GitHub (https://github.com/oxpig/SPACE2).

## Figures and Tables

**Table:** 

**RBD**	receptor-binding domain
**S protein**	spike protein
**NTD**	N-terminal domain
**GP1**	envelop glycoprotein 1
**RMSD**	root mean square deviation
**CDR**	complementarity-determining region

## Data Availability

The original contributions presented in the study are included in the article/Supplementary Material. Further inquiries can be directed to the corresponding author.

## Supplementary material

Supplementary Figures and Tables Data Sheet 1.pdf.

Training set Data Sheet 2.csv.

CoV-AbDab Data Sheet 3.cvs.

Anti-lysozyme antibodies Data Sheet 4.csv
