# Supplementary material for: Improved computational epitope profiling using structural models identifies a broader diversity of antibodies that bind to the same epitope
Source: Front Mol Biosci. 2023 Sep 18;10:1237621. doi: 10.3389/fmolb.2023.1237621 (PMC10544996; doi:10.3389/fmolb.2023.1237621)
Supplement: Supplementary file 3 [file DataSheet1.PDF]

Supplementary Material

1 SUPPLEMENTARY TABLES AND FIGURES

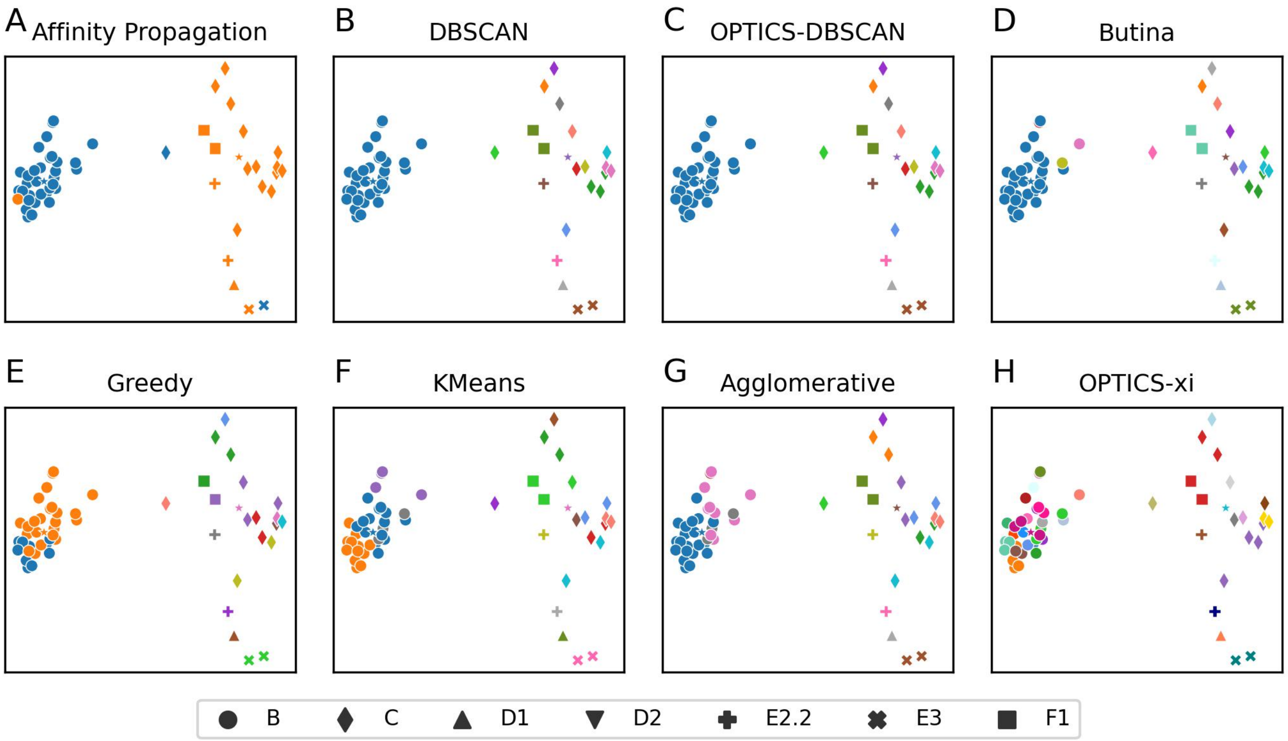

Figure S1. Visualisation of the clustering obtained by the eight algorithms. Principal coordinate analysis of the RMSD matrix for a group of 62 antibodies with identical CDR lengths in the Cao et al. (2023) training set (length CDRH1: 13 residues, H2: 10, H3: 16, L1: 12, L2: 8, L3: 9). The data points corresponding to the antibodies are coloured by the cluster assigned by one of the algorithms (affinity propagation (A), DBSCAN (B), OPTICS-DBSCAN (C), Butina clustering (D), greedy clustering (E), k-means (F), agglomerative clustering (G), OPTICS-xi (H)) using optimal parameters. The marker style indicates the epitope group the corresponding antibody engages.

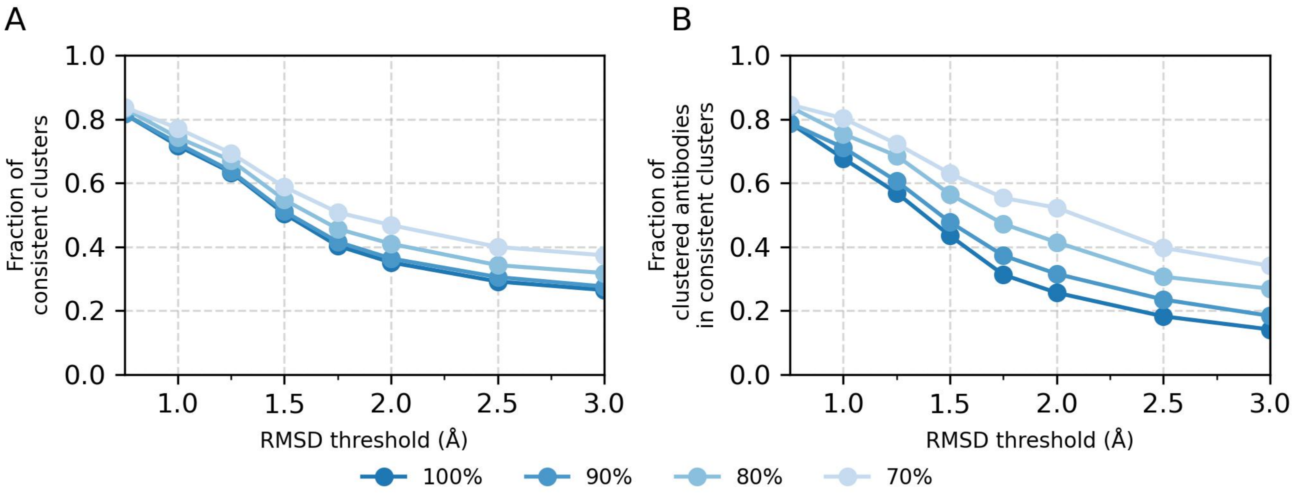

Figure S2. Extent of cluster inconsistency. Accuracy metrics used in this study only capture clusters that exclusively contain antibodies against the same epitope (100% consistency). The two accuracy metrics, (A) the fraction of epitope-consistent clusters and (B) the fraction of clustered antibodies in epitope-consistent clusters, were recalculated considering clusters where at least 90%, 80% and 70% of the members engage the same epitope to be epitope-consistent. This provides a measure of the extent of inconsistency in clusters not captured by the standard performance metrics. A large number of clusters are observed where most antibodies engage the same epitope, but contain a few incorrect antibodies.

Table S1. Performance of agglomerative and OPTICS-xi clustering on train set.

Metric	Agglomerative clustering	OPTICS-xi
Fraction of consistent clusters	0.63	0.59
Fraction of clustered antibodies in consistent clusters	0.57	0.55
Multiple-occupancy clusters	480	584
Antibodies in multiple-occupancy clusters	1446	1615
Antibodies in epitope-consistent multiple-occupancy clusters	823	884
Mean cluster size	3.0	2.7
Max cluster size	28	11

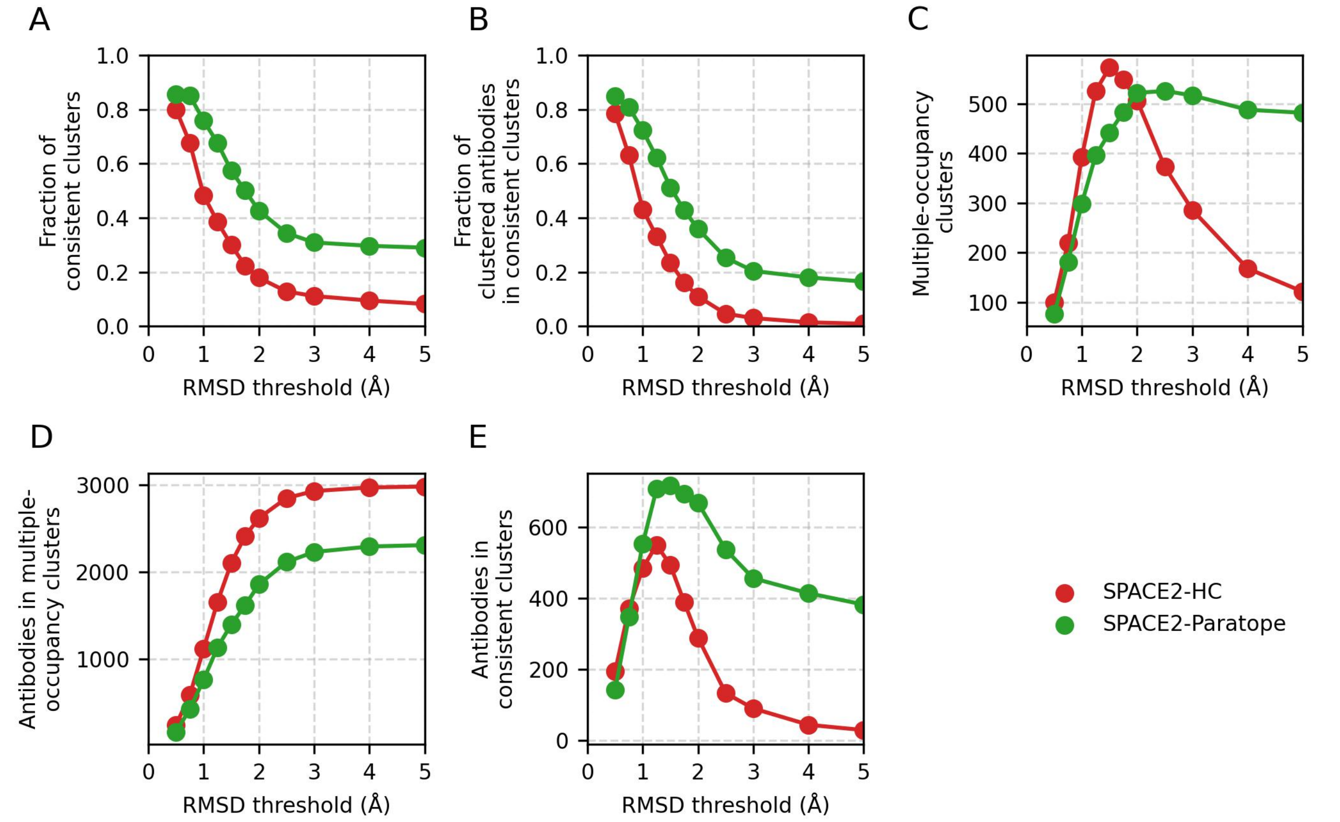

Figure S3. Parameter optimisation of SPACE2 variations. Versions of the algorithm clustering antibodies based on the similarity of heavy chain CDRs (SPACE2-HC) and the similarity of CDRs predicted to contain paratope residues (SPACE2-Paratope) were implemented. As the standard SPACE2 method, both SPACE2-HC and SPACE2-Paratope cluster antibodies using an agglomerative clustering algorithm with a 'complete' linkage criterion. A scan of the RMSD threshold parameter was performed to find optimal values. Optimisation was carried out on the Cao et al. (2023) training set. The values of the five performance metrics are plotted against evaluated threshold values: (A) fraction of epitope-consistent clusters, (B) fraction of clustered antibodies in epitope-consistent clusters, (C) number of multiple-occupancy clusters, (D) number of antibodies in multiple-occupancy clusters, (E) number of antibodies in epitope-consistent multiple-occupancy clusters. The best clustering results are achieved at a threshold value of 1.25 Å for SPACE2-HC and 1.5 Å for SPACE2-Paratope as defined by the maximum number of antibodies in epitope-consistent clusters.

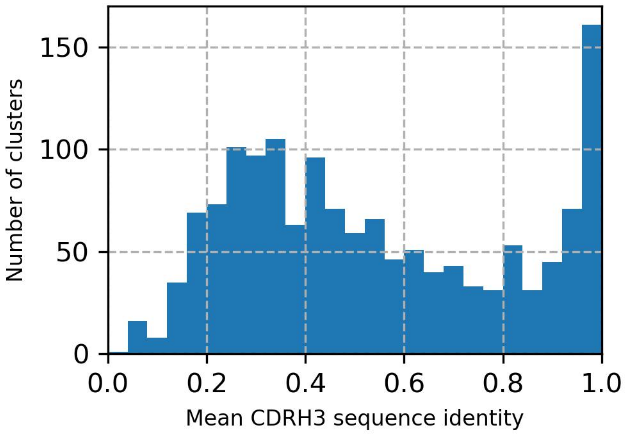

Figure S4. Histogram of the mean CDRH3 sequence identity within domain-consistent SPACE2 clusters from CoV-AbDab. The distribution shows two peaks. The peak close to 1.0 indicates a group of clusters exclusively containing antibodies with almost identical sequences. A second peak at 0.3 indicates the presence of a large number of clusters containing antibodies that are highly diverse in sequence.

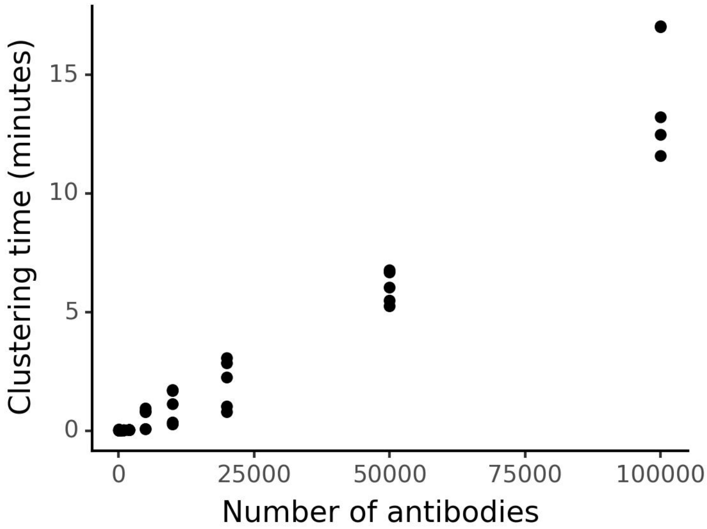

Figure S5. SPACE2 clustering speed. The SPACE2 algorithm functions in four steps: structural modelling of antibodies, grouping by CDR length, computation of the RMSD matrices and agglomerative clustering. Structural modelling with ABodyBuilder2 is currently the rate limiting step, which take around 5 seconds per structure using a Tesla P100 GPU (Abanades et al., 2022). To determine the computational cost of the remaining three steps, we clustered a database of 148,000 paired natural antibody sequences from the Observed Antibody Space database pre-modelled with ABodyBuilder2 (Abanades et al., 2022; Olsen et al., 2022). The graph shows the time required to run the remaining three steps of the clustering code parallelised over 12 CPUs plotted against the number of antibodies to be clustered. These steps scale roughly at $O(n^{1.5})$ with the number of antibodies (n). It is possible to cluster a data set of 10,000 antibodies with SPACE2 within half a day (including all steps of the algorithm). In comparison, clonotyping has a lower computational cost. Our in-house script for Fv-clonotyping takes approximately 2 seconds to cluster 10,000 antibodies on a single CPU.

Table S2. Comparison of standard SPACE2, SPACE2-HC and SPACE2-Paratope performance metrics on the Cao et al. (2023) training set. The best results for each metric are highlighted in bold.

Metric	Standard SPACE2	SPACE2-HC	SPACE2-Paratope
Fraction of consistent clusters	**0.63**	0.36	0.57
Fraction of clustered antibodies in consistent clusters	**0.57**	0.33	0.51
Multiple-occupancy clusters	480	**526**	442
Antibodies in multiple-occupancy clusters	1446	**1655**	1339
Antibodies in epitope-consistent multiple-occupancy clusters	**823**	550	717

Table S3. Comparison of SPACE1 evaluated at different RMSD thresholds on the Cao et al. (2023) training set. The lower threshold leads to better metrics of clustering accuracy. However, a larger threshold improves data coverage and the key metric of antibodies in epitope-consistent multiple-occupancy clusters. The best results achieved by SPACE1 for each metric are highlighted in bold. Values achieved by SPACE2 are shown for comparison.

Metric	SPACE1 0.75 Å	SPACE1 1.25 Å	SPACE2
Fraction of consistent clusters	**0.75**	0.64	0.63
Fraction of clustered antibodies in consistent clusters	**0.68**	0.58	0.57
Multiple-occupancy clusters	251	**314**	480
Antibodies in multiple-occupancy clusters	723	**935**	1446
Antibodies in epitope-consistent multiple-occupancy clusters	494	**538**	823
Fraction of clusters containing >1 VH-clonotypes	0.55	**0.58**	0.55
Mean CDRH3 sequence identity	0.70	**0.67**	0.66

Table S4. In depth comparison of SPACE2 and SPACE1 performance on the Cao et al. (2023) training set. SPACE1 with a 1.25 Å RMSD threshold and SPACE2 as well as an adaptation of SPACE1 using an agglomerative clustering algorithm (complete linkage criterion, 1.25 ÅA RMSD threshold) the default in SPACE2 and an adaptation of SPACE2 using a greedy clustering algorithm (1.25 Å RMSD threshold) the default in SPACE1 were evaluated on the complete data set (all). Additionally, SPACE2 and its adaptation were evaluated on a reduced data set which only included the 2140 antibodies successfully modelled by homology modelling (reduced set).

	SPACE1		SPACE2			
Data set	All		Reduced set		All	
Clustering algorithm	Greedy	Agglomerative	Greedy	Agglomerative	Greedy	Agglomerative
Antibodies modelled	2140	2140	2140	2140	3046	3046
Fraction of consistent clusters	0.64	0.74	0.55	0.63	0.57	0.63
Fraction of clustered antibodies in consistent clusters	0.58	0.7	0.44	0.57	0.44	0.57
Multiple-occupancy clusters	314	292	288	358	389	480
Antibodies in multiple-occupancy clusters	935	802	1109	1104	1451	1446
Antibodies in consistent multiple-occupancy clusters	538	559	485	627	651	823

REFERENCES

Abanades, B., Wong, W. K., Boyles, F., Georges, G., Bujotzek, A., and Deane, C. M. (2022). ImmuneBuilder: Deep-Learning models for predicting the structures of immune proteins. doi:10.1101/2022.11.04.514231

Cao, Y., Jian, F., Wang, J., Yu, Y., Song, W., Yisimayi, A., et al. (2023). Imprinted SARS-CoV-2 humoral immunity induces convergent Omicron RBD evolution. *Nature* 614, 521–529. doi:10.1038/s41586-022-05644-7

Olsen, T. H., Boyles, F., and Deane, C. M. (2022). Observed Antibody Space: A diverse database of cleaned, annotated, and translated unpaired and paired antibody sequences. *Protein Science* 31, 141–146. doi:10.1002/pro.4205
